# Supplementary material for: Puckered and JNK signaling in pioneer neurons coordinates the motor activity of the Drosophila embryo
Source: Nat Commun. 2023 Dec 11;14:8186. doi: 10.1038/s41467-023-43783-1 (PMC10713690; doi:10.1038/s41467-023-43783-1)
Supplement: Supplementary file 1 — Supplementary Information [file 41467_2023_43783_MOESM1_ESM.pdf]

Puckered in pioneer neurons coordinates the motor activity of the *Drosophila* embryo

Katerina Karkali[1,3*], Samuel W. Vernon[2,4], Richard A. Baines[2], George Panayotou[3] and Enrique Martín-Blanco[1 *]

1. Instituto de Biología Molecular de Barcelona (CSIC), Parc Cientific de Barcelona, Baldiri Reixac 10-12, 08028 Barcelona, Spain

2. Division of Neuroscience, School of Biological Sciences, Faculty of Biology, Medicine and Health, University of Manchester, Manchester Academic Health Science Centre, Manchester, M13 9PL, UK.

3. BSRC Alexander Fleming, 34 Fleming Street, 16672 Vari, Greece

4. Present address: EPFL SV BMI UPMCCABE, SV 2511, Station 19, VD 1015, Lausanne, Switzerland.

* Correspondence to: kkabmc@ibmb.csic.es or embbmc@ibmb.csic.es

SUPPLEMENTARY FIGURES

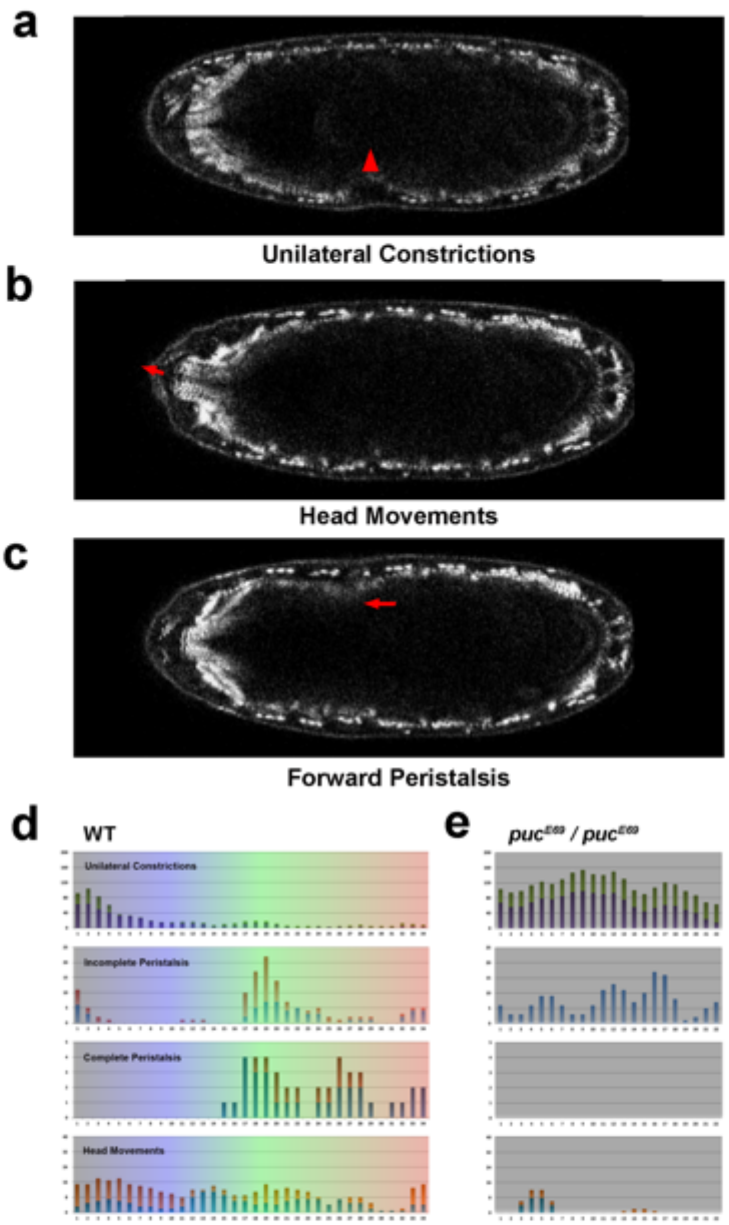

Supplementary Figure 1. Types of embryonic muscle movements

Snapshots from Supplementary Movie 1 displaying mid-plane images of a stage 17 embryo carrying two GFP protein traps expressed at muscle Z-lines (*w; G203, ZCL2144*). Three main types of movement were observed during the muscle coordination process. Red arrows point to the contracting muscle units. Anterior is to the left. Scale bar is 50 µm. a) Unilateral muscle contractions. They can occur at any embryo side and in different segments. b) Head turning and mouth hook pinching. c) Peristalsis. They can be incomplete or complete and develop

forward (in the image) or backward displacements. d) Histograms showing the progression of the muscle coordination process in wild type embryos. Over-imposed colors follow the 5 stages of motor coordination maturation described in c. Muscle movements were distributed in four classes: Unilateral contractions [sum of the contractions occurring at the right (green) and the left (purple) side of the embryo]; Incomplete peristalsis [sum of the incomplete forward (blue) and incomplete backward (red) peristaltic attempts]; Complete peristalsis [sum of the full forward (blue) and backward (red) peristalsis]; and Head movements [sum of head turning (red) and mouth hook pinching events (blue)]. e) Histograms equivalent to d showing the unsuccessful muscle coordination of *puc*^{E69} embryos.

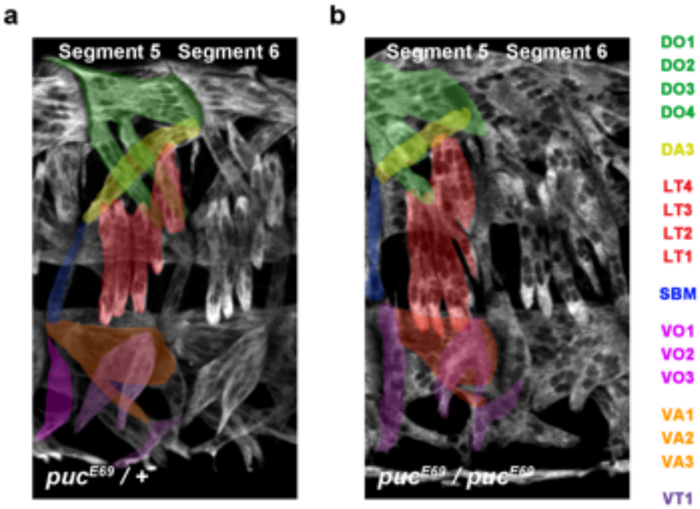

Supplementary Figure 2. Muscles integrity is preserved in *puc* mutants

a and b) Representative images of the Myosin expression pattern, highlighting the full set of somatic muscles, of stage 17 whole mount wild type (*puc*^{E69} / +) (a) and *puc* mutant (*puc*^{E69} / *puc*^{E69}) (b) embryos. The full set of embryonic muscles was traceable in *puc* mutants. Visible external muscles were color coded as indicated.

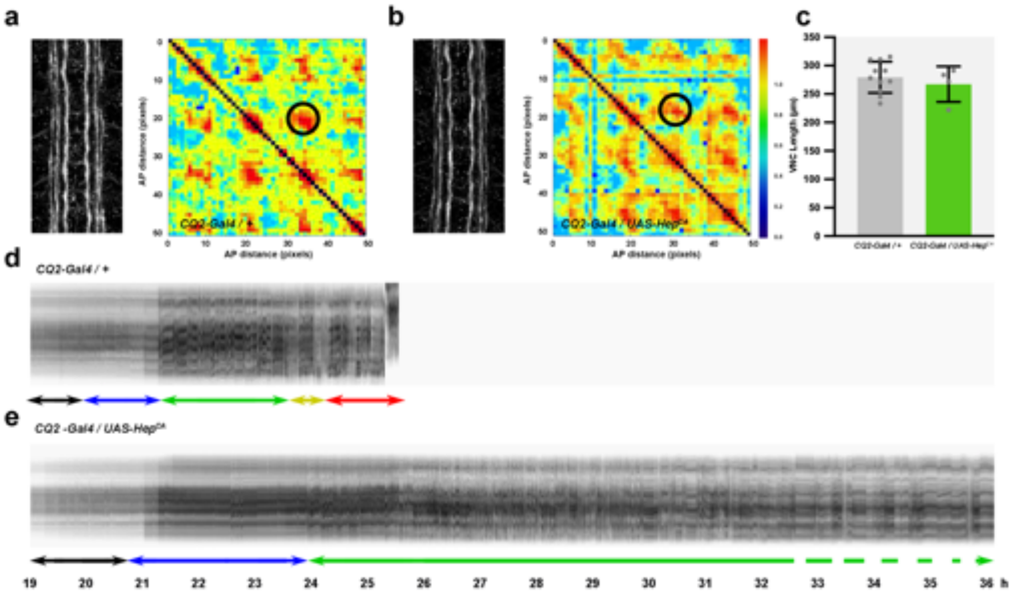

Supplementary Figure 3. JNK hyperactivation in U motorneurons does not affect muscles activities coordination

a and b) Cross-correlation matrices along the AP axis of the VNC of stage 17 embryos stained with Fas 2 (CQ2-Gal4 / + controls (n=4) and CQ2-Gal4 / UAS-Hep^{CA} (n=3). The color-code indicates the correlation level for each possible comparison at each position along the AP axis. The structural organization of the axonal network [3D correlation nodes (black ellipses)] was largely unaffected by Hep^{CA} overexpression in the CQ2 cells (compare a to b). c) Quantification of the VNC length in μm (average and standard deviation) for each condition [a (n=5) and b (n=3)]. Statistically significant differences in length were not detected (p=0.4743). d and e) Representative kymographs displaying muscle activity profiles from 19 to 36 hours AEL of embryos not-expressing (n= 2) (d) or expressing (n= 8) (e) Hep^{CA} in CQ2 motoneurons. The different stages of muscles activities are color coded as in Figure 1. While in the absence of Hep^{CA} a normal pattern of muscle coordination takes place, the overexpression of Hep^{CA} in CQ2 cells arrested the embryos at stage C, showing continuous backward and forward complete peristalsis.

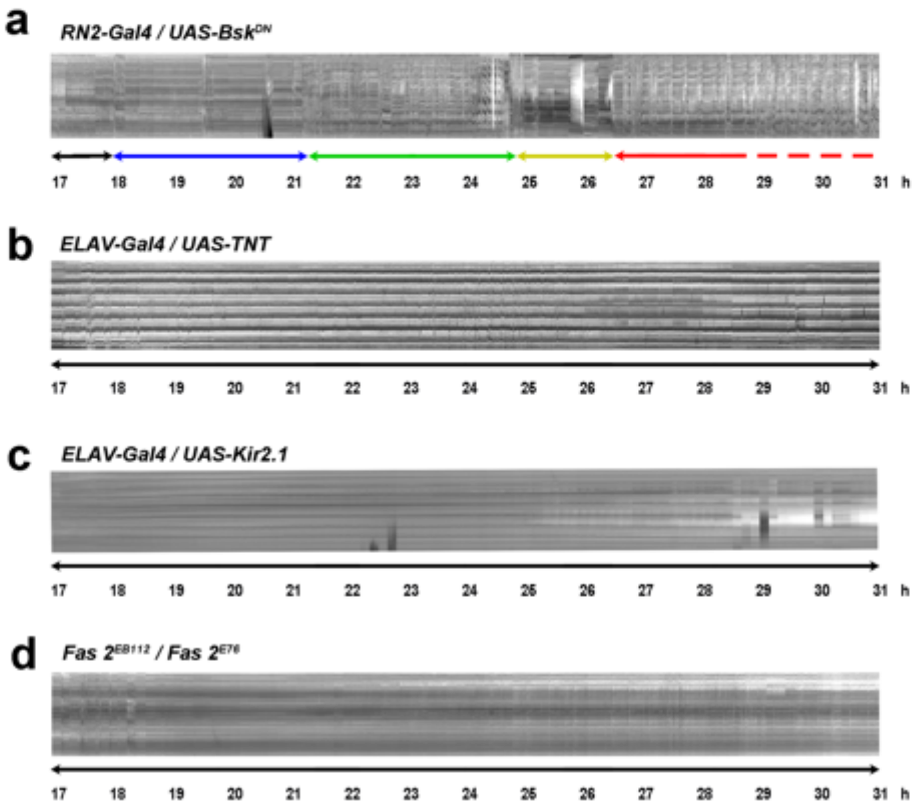

Supplementary Figure 4. Muscle coordination is not affected by a reduction of JNK activity in RN2 cells but abolished by interfering in neurotransmiter exocytosis, neuronal polarity or Fas 2 expression

a to d) Representative kymographs displaying muscle activity profiles from 17 to 31 hours AEL of embryos -expressing Bsk^{DN} in RN2 neurons (n= 4) (a), TNT pan-neurally in ELAV neurons (n= 2) (b), Kir2.1 pan-neurally in ELAV neurons (n= 2) (c) and in an heteroallelic Fas 2 condition (Fas 2^{EB112} / Fas 2^{E76}) (n=2) (d). The different stages of muscles activities are color coded as in Figure 1. The overexpression of Bsk^{DN} in RN2 cells did not affect muscles coordination but resulted in a failure of hatching completion. Conversely, the pan-neural overexpression of TNT or Kir2.1 fully blocked all coordination steps and the embryos remain in an abortive stage A, with limited spontaneous twitches. The same was observed in Fas 2 mutant conditions.

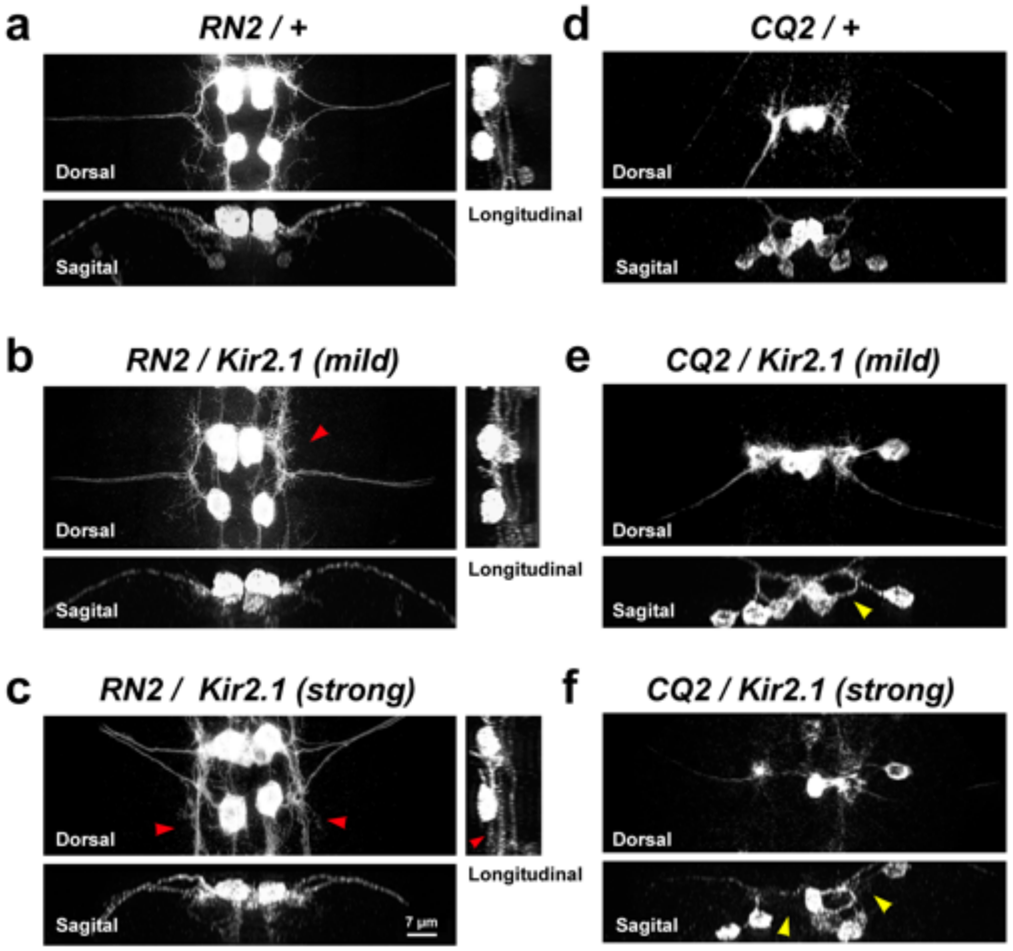

Supplementary Figure 5. Axonal and dendritic landscape of pioneer neurons is altered upon Kir2.1 overexpression

a to f) Single segments (dorsal, sagital and longitudinal (for RN2) views of stage 17 embryos expressing mCD8-GFP (RN2 n=8 and CQ2 n=7) (a and d) and mCD8-GFP and Kir2.1(RN2 n=15 and CQ2 n=11) (b, c, e and f) under the control of the RN2-Gal4 (a to c) and CQ2-Gal4 (d to f) lines monitored live. b and c, and e and f, show, respectively mild and strong phenotypic defects for each condition. Expressivity was variable but penetrance was superior to 80% for both Gal4 lines. Red arrowheads point to altered dendritic arborization pattern in RN2 cells expressing Kir2.1. Yellow arrowheads point to positions void of labelling in CQ2 cells expressing Kir2.1. Anterior is up (dorsal and longitudinal views). Scale bar is 7 µm.

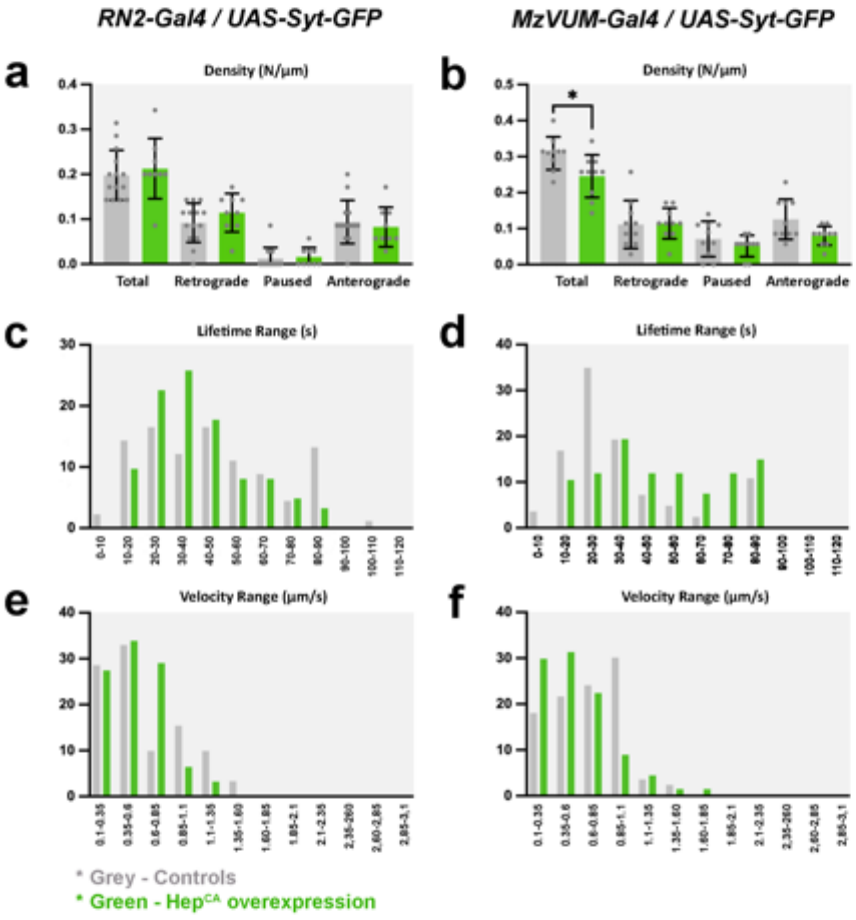

Supplementary Figure 6. Synaptotagmin transport is unaffected by JNK hyper-activation in aCC/RP2 and VUM motoneurons

a and b) Histograms showing synaptotagmin vesicles density and directional motility in control (grey) versus Hep^{CA} expressing (green) aCC/ RP2 (a) and VUM motoneurons (b). (n=9 for RN2 controls, n=9 for RN2 experimental, n=10 for MzVum controls and n=10 for MzVum experimental). Only a slight decrease in total numbers was observed for MzVum between control and JNK over-active neurons (p=0.0194). No significant differences were observed for the rest of the conditions (RN2 total (p=0.9073), RN2 retrograde (p=0.6821), RN2 paused (p=0.9995), RN2 anterograde (p=0.9623), CQ2 retrograde (p=0.9999), CQ2 paused (p=0.8435) and CQ2 anterograde (p=0.1471). c and d) Lifetime range distribution of motile synaptotagmin vesicles in aCC/RP2 (c) and VUM (d) motoneurons. No differences were observed in lifetime between control (grey) and Hep^{CA} expressing (green) embryos. e and f) Mean velocity range distribution comparison of motile synaptotagmin vesicles in aCC/RP2 (e) and VUM (f) motoneurons between control (grey) and JNK gain-of function (green) conditions. For both sets of motoneurons the distribution was unaltered.
