# Supplementary material for: Puckered and JNK signaling in pioneer neurons coordinates the motor activity of the Drosophila embryo
Source: Nat Commun. 2023 Dec 11;14:8186. doi: 10.1038/s41467-023-43783-1 (PMC10713690; doi:10.1038/s41467-023-43783-1)
Supplement: Supplementary file 9 — Reporting Summary [file 41467_2023_43783_MOESM9_ESM.pdf]

nature portfolio

Corresponding author(s): Katerina Karkali & Enrique Martin-Blanco

Last updated by author(s): Nov 13, 2023

Reporting Summary

Nature Portfolio wishes to improve the reproducibility of the work that we publish. This form provides structure for consistency and transparency in reporting. For further information on Nature Portfolio policies, see our Editorial Policies and the Editorial Policy Checklist.

Statistics

For all statistical analyses, confirm that the following items are present in the figure legend, table legend, main text, or Methods section.

n/a	Confirmed	
[ ]	[x]	The exact sample size (*n*) for each experimental group/condition, given as a discrete number and unit of measurement
[x]	[ ]	A statement on whether measurements were taken from distinct samples or whether the same sample was measured repeatedly
[ ]	[x]	The statistical test(s) used AND whether they are one- or two-sided<br>*Only common tests should be described solely by name; describe more complex techniques in the Methods section.*
[x]	[ ]	A description of all covariates tested
[x]	[ ]	A description of any assumptions or corrections, such as tests of normality and adjustment for multiple comparisons
[ ]	[x]	A full description of the statistical parameters including central tendency (e.g. means) or other basic estimates (e.g. regression coefficient) AND variation (e.g. standard deviation) or associated estimates of uncertainty (e.g. confidence intervals)
[ ]	[x]	For null hypothesis testing, the test statistic (e.g. *F*, *t*, *r*) with confidence intervals, effect sizes, degrees of freedom and *P* value noted<br>*Give P values as exact values whenever suitable.*
[x]	[ ]	For Bayesian analysis, information on the choice of priors and Markov chain Monte Carlo settings
[x]	[ ]	For hierarchical and complex designs, identification of the appropriate level for tests and full reporting of outcomes
[x]	[ ]	Estimates of effect sizes (e.g. Cohen's *d*, Pearson's *r*), indicating how they were calculated

Our web collection on statistics for biologists contains articles on many of the points above.

Software and code

Policy information about availability of computer code

Data collection	No software was used
Data analysis	Image analysis and quantification of fluorescence intensity were performed using Fiji 2, 2.14.0/1.54g<br>All statistical analyses were performed using Prism10 Graph Pad Software (Graph Pad Software Inc., La Jolla, CA, USA). In all cases one sample t test or one way ANOVA for multiple comparisons were performed and probability values p<0.05 were considered as significant.<br>The correlation analysis was performed in Matlab R2023a using standard Matlab functions. Full code is provided on Github: https:// github.com/ TimSaunderslab.

For manuscripts utilizing custom algorithms or software that are central to the research but not yet described in published literature, software must be made available to editors and reviewers. We strongly encourage code deposition in a community repository (e.g. GitHub). See the Nature Portfolio guidelines for submitting code & software for further information.

Data

Policy information about availability of data

All manuscripts must include a data availability statement. This statement should provide the following information, where applicable:

- Accession codes, unique identifiers, or web links for publicly available datasets
- A description of any restrictions on data availability
- For clinical datasets or third party data, please ensure that the statement adheres to our policy

The authors declare that all data supporting the findings of this study are available within the article, its supplementary information files and the Source Data file and are also available from the corresponding authors on request

Research involving human participants, their data, or biological material

Policy information about studies with human participants or human data. See also policy information about sex, gender (identity/presentation), and sexual orientation and race, ethnicity and racism.

Reporting on sex and gender	Not Relevant
Reporting on race, ethnicity, or other socially relevant groupings	Not Relevant
Population characteristics	Not Relevant
Recruitment	Not Relevant
Ethics oversight	Not Relevant

Note that full information on the approval of the study protocol must also be provided in the manuscript.

Field-specific reporting

Please select the one below that is the best fit for your research. If you are not sure, read the appropriate sections before making your selection.

☒ Life sciences ☐ Behavioural & social sciences ☐ Ecological, evolutionary & environmental sciences

For a reference copy of the document with all sections, see nature.com/documents/nr-reporting-summary-flat.pdf

Life sciences study design

All studies must disclose on these points even when the disclosure is negative.

Sample size	No sample size calculation was performed. Sample sizes (micrograph materials) were chosen by visual inspection of the data and of a reasonable size to obtain significative statistical values
Data exclusions	Samples of not enough graphical quality or damaged were excluded
Replication	Experiments were repeated at least 3 times and were always reproducible
Randomization	Not relevant. Individual flies/embryos were chosen randomly amongst those with the appropriate markers
Blinding	Most of the presented data is based in graphic information (micrographs). All images were evaluated by at least two authors. Blinding was not relevant

Reporting for specific materials, systems and methods

We require information from authors about some types of materials, experimental systems and methods used in many studies. Here, indicate whether each material, system or method listed is relevant to your study. If you are not sure if a list item applies to your research, read the appropriate section before selecting a response.

Materials & experimental systems

n/a	Involved in the study
☐	☒ Antibodies
☒	☐ Eukaryotic cell lines
☒	☐ Palaeontology and archaeology
☐	☒ Animals and other organisms
☒	☐ Clinical data
☒	☐ Dual use research of concern
☒	☐ Plants

Methods

n/a	Involved in the study
☒	☐ ChIP-seq
☒	☐ Flow cytometry
☒	☐ MRI-based neuroimaging

Antibodies

Antibodies used

IImmunostaining of flat-prepped stage 16 Drosophila embryos was performed using the primary antibodies: mouse anti-Fas 2 (1:100, DSHB, #1D4 anti-Fasciclin II, RRID:AB_528235), and rabbit anti-GFP tag polyclonal (1:600, Thermo Fisher Scientific, # A-11122). Immunostaining of whole mount stage 17 Drosophila embryos was done using a rabbit anti-Drosophila muscle myosin sera 54 at 1:500 dilution (a gift from Dan Kiehart).
The antibodies used for detection were: Goat anti-Rabbit IgG (H+L), Alexa Fluor 488 conjugate (Thermo Fisher Scientific, #A-11008) and Goat anti-Mouse IgG (H+L), Alexa Fluor 555 conjugate (Thermo Fisher Scientific, #A-21422). All secondary antibodies were used in a dilution of 1:600.

Validation

Commercially available antibodies have been extensively validated at the commercial sources and by numerous publications. All other were validated by signal detection in micrographs

Animals and other research organisms

Policy information about studies involving animals; ARRIVE guidelines recommended for reporting animal research, and Sex and Gender in Research

Laboratory animals

Drosophila melanogaster
The following stocks were used:
w[1118]; pucE69LacZ/TM3, twi-GFP 53
w[1118]; P{w[+mC]=UAS-Hep.Act}2 (BDSC #9306)
w[1118]; P{w[+mC]=eve-Gal4.RN2}T, P{w[+mC]=UAS-mCD8::GFP.L}; P{w[+mC]=eve-Gal4.RN2}G, P{w[+mC]=UAS-mCD8::GFP.L} (Dr. Irene Miguel-Aliaga)
w[1118] P{w[+mW.hs]=GawB}MzVUM; P{y[+t7.7]w[+mC] = 10xUAS-IVS-mCD8::GFP} attP40 (Dr. Irene Miguel-Aliaga)
w[1118]; G203:UAS-TNT; ZCL2144 8 (Dr. Matthias Landgraf)
w[1118]; ; ZCL2144 8 (Dr. Matthias Landgraf)
w[1118]; P{UAS-syt.eGFP}2 (BDSC #6925)
w[1118]; P{w[+mC]=UAS-mitoGFP.AP}2 / CyO (BDSC #8442)
w[1118]; P{w[+mC]=UAS-Hsap\KCNJ2.EGFP}7 (BDSC #6595)
w[1118] P{w[+mC]=UAS-bsk.DN}2 (BDSC #6409)
In all cases, unless otherwise stated, embryos of the w1118 strain served as controls.
Aging was not relevant in crosses of adults. Data was retrieved from embryos of different ages as stated in the manuscript

Wild animals

No wild animals were used in the study.

Reporting on sex

Sex- and gender-based analyses were not relevant to a study of embryonic development in flies

Field-collected samples

No field collected samples were used in the study.

Ethics oversight

This study did not require an ethical approval,

Note that full information on the approval of the study protocol must also be provided in the manuscript.
